# Islamic praying changes stress-related hormones and genes

**DOI:** 10.25122/jml-2021-0167

**Published:** 2022-04

**Authors:** Vahid Sobhani, Ehsan Manshadi Mokari, Jafar Aghajani, Boshra Hatef

**Affiliations:** 1. Exercise Physiology Research Center, Lifestyle Institute, Baqiyatallah University of Medical Sciences, Tehran, Iran; 2. Neuroscience Research Center, Baqiyatallah University of Medical Sciences, Tehran, Iran; 3. Department of Molecular Genetics, Faculty of Marvdasht, Islamic Azad University, Shiraz, Iran

**Keywords:** Islam, praying, salivary cortisol, alpha-amylase, IL-6, brain-derived neurotrophic factor

## Abstract

Islamic praying (Namaz) can be considered a mental, spiritual, and physical practice. The study aimed to investigate the early effect of Namaz on stress-related hormones and the expression of stress-induced genes such as IL6 and BDNF. Eighty-three healthy women and men who continually practice Namaz participated in the study. The saliva samples were taken before and after Namaz to measure cortisol and alpha-amylase hormone levels. Also, to evaluate the expression of BDNF and IL6 genes, 11 specimens were selected randomly. Based on baseline sampling, the participants were classified into three groups: cortisol levels lower than 5, between 5–15, and upper than 15 ng/ml. The results indicated that cortisol significantly increased and decreased in the first and third groups after Namaz, respectively. In addition, the increase of alpha-amylase also occurred in subjects with a low baseline level of its concentration. Regarding genetic expression examination, there was a significant decrease in BDNF gene expression after the Namaz. In addition, the change of cortisol and alpha-amylase hormones after Namaz related to the baseline level changed to approach the optimal range after Namaz. These findings were reported for the first time and need more studies.

## Introduction

There are more than 1.2 billion Muslims worldwide [1]. The Muslim should perform a praying task by introducing Salat in Arabic and Namaz in Turkish and Persian. It involves various body postures through which different specific and meaningful verses have to be recited [2]. Namaz is a kind of physical activity implementing particular positions, including standing, bowing, prostration, and sitting [3, 4]. Namaz performed five times a day at specific times is a unique way of worshiping God among Muslims to improve the physical, mental and psychological state of mind in alertness and concentration [3, 4]. Considering the frequent concentration, repetition of specific phrases, and focus on God's worship, Namaz can be introduced as an evolving meditative exercise [1, 5].

Stress impairs mental health and is a normal part of everyday life, usually referred to as an unfavorable condition in which the outcome is not clear for the individual. At the same time, it can be created through some internal and external causes [6]. When stress is perceived, the hypothalamic-pituitary-adrenal axis (HPA) with the release of cortisol activates. Moreover, the sympathetic adrenal medullary (SAM) axis, whose markers include heart rate and alpha-amylase hormone variations, activates stress-related behaviors. As a result, the person's consciousness rises, and the level of energy, cardiovascular power, and immunity increase to provide an appropriate response to the stressor. After that, the body returns to the base state through homeostatic mechanisms [7]. However, a high level or repetitions of stress can disrupt the body's homeostasis and affect the cardiovascular, immune, and reproduction system [8].

Meditation is generally defined as a kind of mental exercise to improve individual mental capacities. It is a self-regulating action to improve mental and emotional control [9, 10]. There are many studies on the effects of meditation. Lee *et al.* [11] found that heart rate, respiration rate, and systolic blood pressure decreased significantly during Qi-training. Raichur and colleagues [12] explained the effects of meditation exercises on breathing change. Travis showed that transcendental meditation decreased the breathing rate and increased respiratory sinus arrhythmia amplitudes and EEG amplitude [13]. Yoga training improves life quality, symptoms of depression, anxiety, stress, and post-traumatic stress disorder [14, 15]. Decades of research have linked mediation-based approaches to positive psychological and health outcomes in clinical and non-clinical groups [16, 17]. Recently, researchers have tried to understand the biological mediation mechanisms of mental improvement in well-being, including changes in physiology, neurology, immunity, and genomics [18]. The meta-analysis indicated the effects of mindfulness-based interventions (MBIs) for 6–10 weeks on salivary cortisol levels in healthy subjects. The studies suggested that MBIs might have a little beneficial effect on cortisol secretion [19]. On the other hand, few studies showed that Namaz increases parasympathetic and the alpha band of brain-power frequency, which results in relaxation and concentration in contrast with stress effects [2, 4, 5, 20]. 

Brain neurotrophic factor (BDNF) is a stress response system and a modulator of brain neurons that promotes neurons' development, survival, and flexibility in the central and peripheral nervous systems. This protein enhances acute stress [21]. The reduction of BDNF levels is associated with chronic psychiatric and neurological disorders, including anxiety and depression [22, 23]. The BDNF gene expression increased after three months of yoga training [24]. BDNF increases in acute stress and decreases in chronic disorders related to stress. 

Another biologically essential and related aspect of psychological health is the inflammatory pathway. Research over the past 20 years showed that the physical states of inflammatory protein are associated with anxiety disorders [25]. Psychosocial stress can cause chronic inflammatory states [26–28]. For example, IL-6 is stimulated along with inflammatory protein pathways through psychological stress, fever, or response to the acute phase. IL-6 is also known as a myokine, which has anti-inflammatory effects when stimulated by exercising [29]. Jang *et al.* showed that mind-body training increased IL-10 under specific conditions, which may regulate the stress response and relationship between the immune and nervous systems [30]. Nevertheless, no study was done to assess the biological effect of Namaz on stress-related factors. 

This study aimed to investigate the acute effects of Namaz on the activity of the HPA and SAM axis and changes in molecular stress markers such as gene expression of IL6 and BDNF. The hypothesis was that Namaz has a significant effect on biological markers of stress.

## Material and Methods

### Participants

Eighty-three volunteers participated in this study. Forty-seven men were 44±14 years old, and 36 women were 34±9 years old. Volunteers were examined based on some entry requirements, including the sustained practice of Namaz, absence of systemic diseases, no use of drugs and psychiatric drugs, and the lack of regular exercise.

### Procedure

The time to visit participants was between 10:00 am and 3:00 pm. The volunteers were asked not to eat anything for an hour before beginning the test. They also washed their mouths before the test and performed their ablution before prayer. Then, about 0.5 ml of saliva sample was taken after filling out a questionnaire concerning personal characteristics. Next, they were asked to perform four rakaats of the actual Namaz. After Namaz, a saliva sample was retaken. The primary outcome measures were the concentration of cortisol and alpha-amylase hormones in saliva, and secondary outcome measures were the change of BDNF and IL-6 gene expression.

### Hormonal measurement

Saliva samples were stored in a -80°C freezer and were analyzed using the ELISA method, with a specific salivary cortisol kit from ZellBio Co. Germany. The proposed normal range (5–21.5 ng/ml) was used to measure the cortisol level through the enzymatic assay and create a dilution of 100 times with the help of the 0bt300 BIOS system device.

### Quantitative Real-Time Reverse Transcription Polymerase Chain Reaction (RT-PCR)

Eleven participants were randomly selected, and their saliva samples were genetically examined for BDNF and IL-6 gene expression. To investigate gene expression, samples were immediately transferred to the laboratory after sampling with cold cargo and stored in a -80°C freezer until the tests. First, extraction of RNA from saliva was performed using the GENE ALL extraction kit manufactured in Korea. Then, the sample was extracted to evaluate the quality of extraction, NanoDrop, and its concentration. The RNA absorption ratio was measured at 260–280 nm wavelengths. To synthesize cDNA, we used the samples with an absorbance at 260 and 280 nm within the range of 1.8–2.2. The extracted specimens were electrophoresed for final confirmation, where 18S and 28S bands were observed. We used ABI (Applied Biosystems) kit without inhibiting RNase. In the next step, to design the appropriate primers, DNA sequences of genes were first taken from the GenBank, and then primers were designed on the Primer3 Website. The best gene area for these primers was determined. To ensure the correct sequence of primers and avoid connecting them to non-specific sequences in other parts of the genome, the utilized primers were BLASTPRIMER [31]. The sequence selected for the BDNF forward primer and the reverse primer were 5`-AGCCTTCATGCAACCAAAGT-3` and 5`-TCGGGTTAGTCTCTGGGGAT-3`) respectively. Also, the primer's sequence for IL6 for the forward primer was 5`-GTCAGGGGTGGTTATTGCAT-3`, and the reverse primer was 5`-AGTGAGGAACAAGCCAGAGC-3`. Then, we made a mixture of primers with a concentration of 10 pmol. There was a primer for the BDNF gene and a primer for the IL6 gene. Inside a microtube, 1.5, 5 Landa from the BDNF forwarder primer, and 5 Landa from the BDNF reverse primer were added with 90 Landa DNase free water. After that, ten μl of Master, one μl of primer, five μl of water, and 4 μl of the sample were added to each microtube 0.2. The samples were tested on a real-time device (Qia gene Rotorgene Q, made in America).

### Statistics

The normal distribution of variables was confirmed by the S-K test. Individuals were divided into three groups based on the salivary cortisol base level, including less than 5, 5–15, and more than 15 ng/ml, to compare the effect of Namaz on the level of salivary cortisol. First, the chi-square test was used to evaluate the distribution of gender between the groups. Then, the effect of Namaz on cortisol changes was examined considering the effect of the base level of cortisol, using two-way mixed-model ANOVA and the test of Bonferroni for pair-wised comparison. Secondly, subjects were divided into two groups of minor and more than 100,000 units per liter in a salivary to analyze the salivary alpha-amylase hormone. Then, the effect of Namaz on alpha-amylase changes was investigated by running the two-way mixed model ANOVA and considering the effect of the alpha-amylase base level. Finally, to determine the expression of IL6 and BDNF genes, their values were calculated through a comparison with the GAPDH ΔCT base gene. Then, paired t-test comparison was performed between ΔCT before and after Namaz. The SPSS version 23 was used for analysis, and P <0.05 was considered significant.

## Results

### The effect of Namaz on salivary cortisol

**Figure 1.**
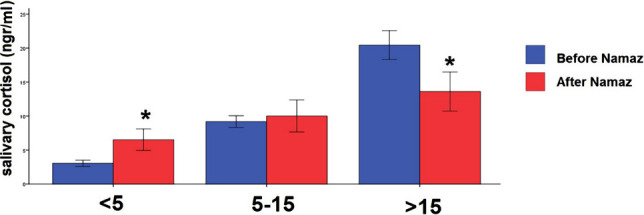
Mean and CI 95% of the salivary cortisol level before and after Namaz in three groups classified according to the cortisol's initial concentration. * – P<0.0005.

After dividing participants into three groups based on the level of cortisol, the distribution of women and men in these groups was as follows: group 1 (cortisol levels below 5 ng/ml) included 12 males and 10 females; group 2 (cortisol levels ranging from 5 to 15 ng/ml) included 24 males and 20 females; and group 3 (cortisol levels above 15 ng/ml) consisted of 11 males and 6 females. There was no significant difference in gender distribution between these three groups. The results of the two-way mixed model ANOVA indicated that the effect of grouping was significant (F (1,79)=16.44, sig=0.000001). After Namaz, cortisol increased significantly in the first group and diminished significantly in the third group (Figure 1).

### The effect of Namaz on salivary alpha-amylase

**Figure 2.**
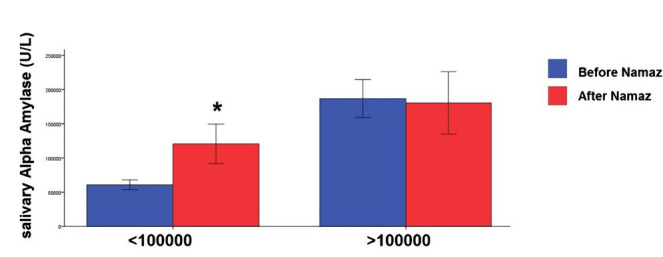
Mean and CI 95% of the salivary alpha-amylase levels before and after Namaz in two groups classified according to the initial concentration of α-amylase. * – P<0.02.

The subjects were divided into two groups based on the level of alpha-amylase. We had the following distribution of men and women: group 1 (alpha-amylase levels less than 100,000 units per liter) included 22 males and 21 females. Group 2 (alpha-amylase levels greater than 100,000 units/liter) consisted of 25 males and 15 females. There was no significant difference in gender distribution between groups (Figure 2). However, the results of the two-way mixed model ANOVA suggested that the effect of grouping was significant (F (1.83)=6.31, sig=0.014), showing a significant increase in alpha-amylase in the first group after Namaz (Figure 2).

### The effect of Namaz on the expression of genes

**Figure 3.**
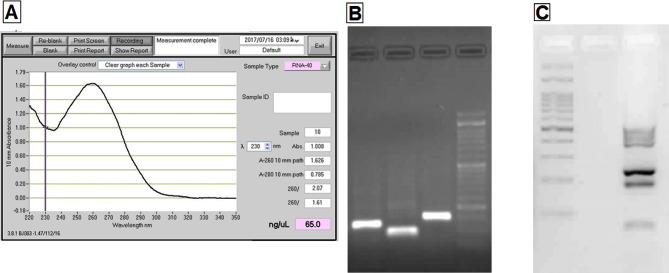
A – Nano-drop Test Diagram. Horizontal Electrophoresis Systems. B – Represents the function of primers on gel electrophoresis. C – RNA extraction accuracy.

After RNA extraction, the sample's concentration and non-contamination were first studied by performing a nano-drop test. The value of the absorbance ratio of 260/280 nm above 2 indicates the correct extraction of RNA and the absence of contamination in the specimens. The sample concentration was 65 ng/uL (Figure 3A). Then, the PCR method and horizontal electrophoresis systems were used to show the performance's accuracy and the absence of DNA (Figure 3B and 3C).

**Figure 4.**
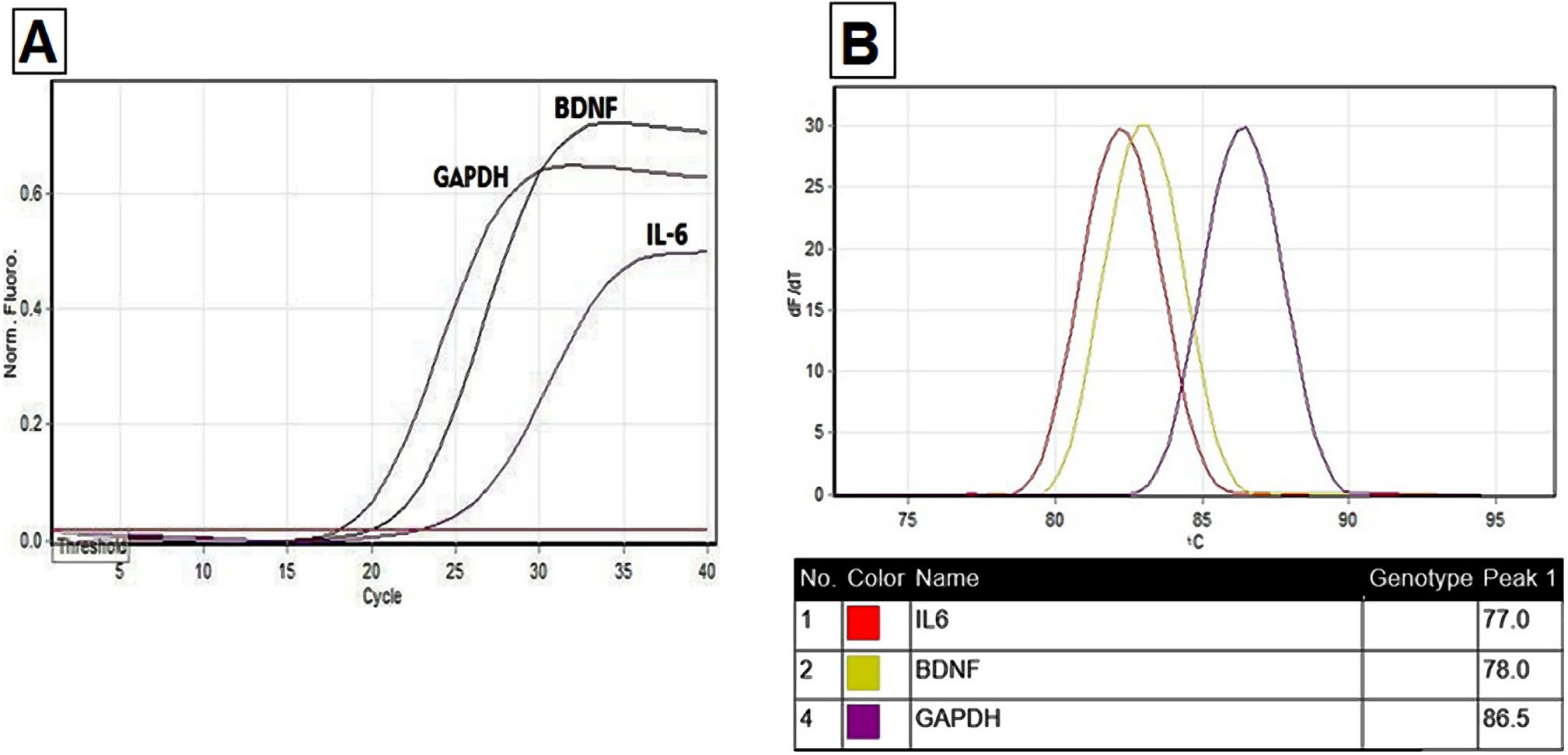
A – Diagram of the expression process of the examined genes. B – The melting chart shows the accuracy of the primer.

**Figure 5.**
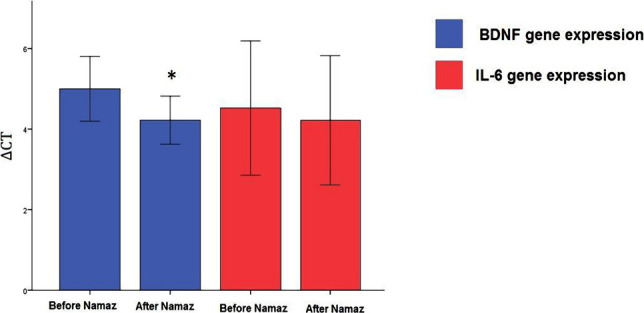
Mean and CI 95% of ΔCT of the expression of BDNF and IL-6 genes before and after Namaz.

According to Figure 4A, it can be concluded that the logarithmic increase in the absorption rate is indicative of the correctness of the reaction. Also, the lack of additional peaks confirms the absence of non-specific bands. The courier is entirely a bell tube that confirms the absence of smear in the reaction process (Figure 4B). The results of comparing ΔCT of the expression of BDNF and IL-6 genes before and after Namaz revealed that the expression of the BDNF gene decreased significantly (Figure 5). The ΔCT of the expression of the IL-6 gene did not change significantly.

## Discussion

To the best of the authors' knowledge, this study was the first to investigate the acute effect of Namaz on the level of salivary cortisol and alpha-amylase hormones and variations of the IL6 and BDNF gene expression.

Praying is the most excellent worship and the most important order of the divine prophets. It is an integral part of all divine religions, performed specifically in every religion. Many psychologists found that praying and having faith in religion could help individuals overcome the anxiety, depression, and fear of many underlying illnesses [32, 33].

Cortisol is a marker of HPA activity, so its high level, more than the normal range, is a sign of acute stress and its low level is a sign of chronic stress like post-traumatic stress disorder (PTSD) [34, 35]. Accordingly, we classified the pre-Namaz levels of cortisol into three groups to investigate changes in cortisol levels. Interestingly, our results revealed that Namaz decreased the cortisol for the higher concentration group and increased it for the lower than normal concentration range. However, it did not significantly affect the group's cortisol level with a normal range. A similar pattern in both groups occurred for the alpha-amylase enzyme. Notably, these exciting results have not been presented before. Since cortisol has a critical effect on the metabolic function, brain activity, and immune system, and its level is changed during a day, its optimal daily level is essential to maintain the body's optimal performance [34, 35]. Then, we could conclude that Namaz, with all specifications, can play a survival role to restore the optimal level of cortisol in a short time (around 5 minutes).

The results of BDNF gene expression showed that the expression of this gene decreased significantly after Namaz. Several animal studies showed that the BDNF protein and RNA expression increased after acute stress, even in the submandibular gland that released saliva [36, 37]. Although the BDNF decreased in chronic stress and depression [23], the acute increase is not good news. The acute decrease of BDNF expression after Namaz that is repeatedly perceived might have a compensatory or restoring function to decline the effect of acute stress and decrease the long-term effect of stress on the normal expression of BDNF. Some studies showed several beneficial effects of Namaz on mental and physical health, such as relaxation and wellness, that confirmed the current study results [4, 20, 38, 39]. 

Similar studies showed that meditation and yoga intervention for a long time reduces stress and improves mental health [40–44]. For example, one study found that 15 minutes of hatha yoga significantly reduces employees' psychological and physiological markers of stress [45]. Regarding mood, previous studies showed that a hatha yoga session improves POMS scores in psychiatric patients [46] and reduces mental stress in healthy university students [44]. Some of the benefits of a variety of mental mediations are: increasing blood flow to the brain, improving blood oxygenation, increasing brain neurotrophic factor, modulating the HPA axis, reducing acute stress, regulating the muscular system, increasing gamma-amino-butyric acid, or a combination of the mentioned effects [47, 48].

In summary, two significant and remarkable findings emerged from the present study. Notably, Namaz, done in four cycles (Rakaat) that did not take more than five minutes, had some significant hormonal and genetic effects. The salivary cortisol level increased after Namaz in the participants whose cortisol level was lower than the normal range. On the other hand, cortisol concentration decreased in participants with a higher than average cortisol level. Simultaneously, no difference was observed in the subjects with an average level at baseline. The same pattern was seen for the alpha-amylase hormone. Besides, the real-time study showed that the BDNF expression decreased after Namaz. The study's limitations were the absence of a control group who did not pray and the low number of samples in the genetic research because of the limited financial support. Future studies considering the long-term effect of Namaz and comparing it with meditation in different groups are suggested. Given that the number of depressed people is rising due to increasing pressures from social and environmental changes and increasing physical illnesses [49], performing mental exercises such as Namaz is highly recommended to maintain health.

## Conclusion

For the first time, the findings of this study showed that the immediate effect of Namaz on the hormones and genes related to stress improves. Overall, taking the findings of this study into account, we can conclude that Namaz plays an influential role in the peace of mind and body via modulation of stress response even at the gene expression level.

## Acknowledgments

### Conflict of interest

The authors declare no conflict of interest.

### Ethical approval

The study was approved by the Ethics Committee of the Baqiyatallah University of Medical Sciences, Tehran, Iran (IR.BMSU.REC.1397.190)

### Consent to participate

All volunteers signed a written consent letter before participating in the study. 

### Funding

This study was funded by Imam Sadiq (AS) Research Center.

### Authorship

BH and VS contributed to conceptualizing. JA, EMM and BH contributed to the methodology. All authors contributed to writing the original draft and editing the manuscript. All authors contributed to data collection. B.H. contributed to data curation. V.S. and E.M.M. contributed to data analysis.
